# Telehealth Use During the COVID-19 Pandemic Among Veterans and Nonveterans: Web-Based Survey Study

**DOI:** 10.2196/42217

**Published:** 2023-09-08

**Authors:** Holly E Shoemaker, Alistair Thorpe, Vanessa Stevens, Jorie M Butler, Frank A Drews, Nicole Burpo, Laura D Scherer, Angela Fagerlin

**Affiliations:** 1 Department of Population Health Sciences Spencer Fox Eccles School of Medicine University of Utah Salt Lake City, UT United States; 2 Salt Lake City VA Informatics Decision-Enhancement and Analytic Sciences (IDEAS) Center for Innovation Salt Lake City, UT United States; 3 Department of Internal Medicine, Division of Epidemiology, Spencer Fox Eccles School of Medicine, University of Utah Salt Lake City, UT United States; 4 Geriatrics Research, Education, and Clinical Center VA Salt Lake City Health Care System Salt Lake City, UT United States; 5 Department of Biomedical Informatics, University of Utah Salt Lake City, UT United States; 6 Division of Geriatrics, Department of Internal Medicine, University of Utah Salt Lake City, UT United States; 7 Department of Psychology, College of Social and Behavioral Science, University of Utah Salt Lake City, UT United States; 8 Research Operations, American Heart Association Dallas, TX United States; 9 University of Colorado School of Medicine Aurora, CO United States; 10 VA Denver Center for Innovation Denver, CO United States

**Keywords:** telehealth, telemedicine, veterans, COVID-19, health care, nationwide web-based survey, underserved population, accessible health care, health inequality, health care disparity

## Abstract

**Background:**

In the first year of the COVID-19 pandemic, studies reported delays in health care usage due to safety concerns. Delays in care may result in increased morbidity and mortality from otherwise treatable conditions. Telehealth provides a safe alternative for patients to receive care when other circumstances make in-person care unavailable or unsafe, but information on patient experiences is limited. Understanding which people are more or less likely to use telehealth and their experiences can help tailor outreach efforts to maximize the impact of telehealth.

**Objective:**

This study aims to examine the characteristics of telehealth users and nonusers and their reported experiences among veteran and nonveteran respondents.

**Methods:**

A nationwide web-based survey of current behaviors and health care experiences was conducted in December 2020-March 2021. The survey consisted of 3 waves, and the first wave is assessed here. Respondents included US adults participating in Qualtrics web-based panels. Primary outcomes were self-reported telehealth use and number of telehealth visits. The analysis used a 2-part regression model examining the association between telehealth use and the number of visits with respondent characteristics.

**Results:**

There were 2085 participants in the first wave, and 898 (43.1%) reported using telehealth since the pandemic began. Most veterans who used telehealth reported much or somewhat preferring an in-person visit (336/474, 70.9%), while slightly less than half of nonveterans (189/424, 44.6%) reported this preference. While there was no significant difference between veteran and nonveteran likelihood of using telehealth (odds ratio [OR] 1.33, 95% CI 0.97-1.82), veterans were likely to have more visits when they did use it (incidence rate ratio [IRR] 1.49, 95% CI 1.07-2.07). Individuals were less likely to use telehealth and reported fewer visits if they were 55 years and older (OR 0.39, 95% CI 0.25-0.62 for ages 55-64 years; IRR 0.43, 95% CI 0.28-0.66) or lived in a small city (OR 0.63, 95% CI 0.43-0.92; IRR 0.71, 95% CI 0.51-0.99). Receiving health care partly or primarily at the Veterans Health Administration (VA) was associated with telehealth use (primarily VA: OR 3.25, 95% CI 2.20-4.81; equal mix: OR 2.18, 95% CI 1.40-3.39) and more telehealth visits (primarily VA: IRR 1.5, 95% CI 1.10-2.04; equal mix: IRR 1.57, 95% CI 1.11-2.24).

**Conclusions:**

Telehealth will likely continue to be an important source of health care for patients, especially following situations like the COVID-19 pandemic. Some groups who may benefit from telehealth are still underserved. Telehealth services and outreach should be improved to provide accessible care for all.

## Introduction

The COVID-19 pandemic led to unprecedented disruptions and delays in health care delivery due to safety concerns [1,2]. Health care delays can result in increased morbidity and mortality from otherwise preventable or treatable conditions and may be responsible for some of the excess deaths observed during the pandemic, which are not due to COVID-19 infection [3,4].

Telehealth—the provision of health care remotely via information and telecommunications technology [5]—was rapidly adopted as a safe alternative to in-person visits early in the pandemic [6-8]. Data from 4 large national telehealth providers show an increase of 154% during the last week of March 2020 compared to 2019 [9]. In addition to reduced exposure to COVID-19, the increase in telehealth use benefitted providers and patients through improved access to care, reduced cost, reduced use of personal protective equipment, and convenience [10,11].

Telehealth is a promising approach to address the delays or gaps in access to care, particularly for the more than 50% of Americans and 65% of veterans experiencing chronic conditions who require frequent health care visits and are at higher risk of severe outcomes from COVID-19 [12-14]. However, the rapid adoption of new technologies leaves little time to address gaps in patient comfort and access, which may have limited the ability of telehealth to fully mitigate delays in care among patients who are chronically ill.

Unfortunately, our understanding of patient experiences of telehealth during the COVID-19 pandemic remains limited. Previous research primarily used electronic health records to identify the characteristics of telehealth users, but patient experiences and patterns of usage across health care systems are poorly understood [15-20]. To address this gap, we conducted a digital survey of US adults (veterans and nonveterans) during the COVID-19 pandemic to understand the use of telehealth during the first year of COVID-19, which may help direct the future use of telehealth outside the COVID-19 experience. These populations may have had distinct experiences during the pandemic. The veteran population often has different characteristics compared to the general population [21] and may use a unique, specialized health care network in the form of the Veterans Health Administration (VA) [22]. As our study examines health care both inside and outside the VA, this provides the opportunity to observe differences resulting from using telehealth inside and outside of the VA. We assessed the characteristics of users and nonusers of telehealth, the barriers to use of telehealth, and whether the VA’s longstanding use of telehealth increased the likelihood of veterans using telehealth compared to nonveterans.

## Methods

### Recruitment

Participants were recruited to take part in a nationwide study through Qualtrics web-based panels. [23] Qualtrics web-based panels is a commercial survey sampling and administration company with existing pools of potential participants who have agreed to be solicited for participation in survey studies. For this study, Qualtrics web-based panels sent eligible participants an email invitation to take part in the study. Respondents opted to take part in the study by clicking a survey link which directed them to the survey page. Respondents were compensated for taking part in the study depending on how they were recruited and what they selected as their reward. Rewards may have included SkyMiles, points at their favorite retail outlet, gift cards, or cash.

Surveys were conducted in 3 waves using the same participants at each wave. Wave 1 was administered digitally between December 2 and December 27, 2020; wave 2 between January 21 and February 6, 2021; and wave 3 between March 8 and March 23, 2021.

### Ethics Approval

This study was reviewed and approved by the institutional review board of the University of Utah and Research and Development Committee at the VA Salt Lake City Health Care System (IRB_00133198, categorized as exempt). Participants who completed the survey too quickly (>2 SDs faster than the median completion time), failed a reCAPTCHA test, or took more than 24 hours to complete were excluded. Results from the first wave are presented here, as results were similar across all 3 waves.

### Procedures and Methods

Each wave of the survey consisted of questions on respondents' current health behaviors, well-being, health care experiences, and attitudes regarding the COVID-19 pandemic. Telehealth use, frequency, and perceptions were assessed at each wave. Telehealth use was assessed through the question “Have you received any care over the phone or through video chat, instead of in-person visits, since the start of the COVID-19 pandemic?” with 2 options (0=no, I have not received telehealth; 1=yes, I have received telehealth). In waves 2 and 3, the question referenced the time since the last survey and time in the last month, respectively, instead of since the pandemic’s start. The number of telehealth visits was also assessed. Preference for telehealth was measured using a 5-point scale (1=much prefer in person; 5=much prefer telehealth). Demographic questions included age, gender, race and ethnicity, number of comorbid health conditions, and where they reside (ie, US region, urban or rural status, and size of city). Additional characteristics measured include job change due to COVID-19, insurance status, and health care source (primarily VA, non-VA, or combination). Finally, we queried respondents regarding their reasons for telehealth use or nonuse, preference for in-person versus telehealth visit, family member telehealth use, health care source, and general difficulty of getting health care. All surveys may be viewed at [24]. Questions used in this study may also be viewed in Multimedia Appendix 1.

### Analysis

Analyses were conducted in R (version 4.0.2; R Core Team) [25]. Respondent characteristics were summarized with descriptive statistics (counts and percentages). The relationship between respondent characteristics and telehealth use (binary yes or no) and visits (count 0-10+) was assessed through a regression model. Health care use data are notoriously skewed, with a large proportion of patients reporting no visits [26]. To address this issue, we implemented a 2-part model to account for zero skewed data, first by modeling the probability of having any visits, followed by a truncated count model for those with 1 or more visits. The zero distribution used a binary logit link (1 or more visits compared to none), while the truncated count portion of the model used a negative binomial distribution to account for overdispersion. In addition, we conducted a supplementary analysis where we ran this model on the veteran and nonveteran population separately, which can be found in Multimedia Appendix 1.

Due to a survey delivery error, education status was missing for a large portion of respondents (638/2085, 31%) at baseline. To help account for this, an average of 3 numeracy questions, which assess the ability to understand and work with numbers, as defined by McNaughton et al [27], was added to the model as a proxy. Model results from the first wave are presented here. The results were similar across all waves (data not shown).

## Results

### Survey Response

There were 2085 total responses to the first wave, of which 1060 were veterans and 1025 were nonveterans (see Table S1 in Multimedia Appendix 1).

As shown in Table S1 in Multimedia Appendix 1, a large majority of veterans were male (975/1060, 92%), while more than half of nonveterans were female (551/1025, 53.8%). Veterans tended to be older than nonveterans, with a median age range of 65-74 years compared to 45-54 years. A majority of the respondents identified as White or European American (879/1060, 82.9% of veterans and 842/1025, 82.1% of nonveterans), with Black or African American as the second most common reported race (129/1060, 12.2% of veterans and 133/1025, 13% of nonveterans). Median income among veterans (US $75,000-US $99,999) was higher than nonveterans (US $60,000-US $74,999). A majority of veterans reported primarily getting their health care from non-VA sources (781/1060, 73.7%). Veterans who reported receiving health care from non-VA or an equal mix of VA care had higher median income compared to those who reported receiving health care primarily at the VA (US $75,000-US $99,999 vs US $50,000-US $59,999). Women accounted for a higher proportion of veterans who received health care from the VA than the general veteran group as well. Only 7.9% (84/1060) of the veteran group is female, but they make up 17.2% (29/169) of those who received health care primarily at the VA.

Overall, 898 (43.1%) respondents reported using telehealth since the pandemic began, including 474 (44.7%) veterans and 424 (41.4%) nonveterans (Table 1).

Among those who used telehealth, the median number of visits for both groups was 2 (IQR 1-3). The median age range of those who used telehealth was 65-74 years among veterans and 35-44 years among nonveterans.

Veterans and nonveterans who used telehealth had similar gender proportions to the overall group; 91.6% (434/474) of veterans and 45% (191/424) of nonveterans were male. Racial groups were also similar to the overall population. Most telehealth users identified as White or European American (716/898, 79.7% of telehealth users), followed by Black or African American as the second most common reported race (137/898, 15.3% of telehealth users). A larger proportion of telehealth users reported having 2 or more comorbidities than nonusers (483/898, 54.5% compared to 369/1187, 31.4%), and this number was higher in veteran telehealth users when compared to nonveterans (289/474, 61.4% vs 194/424, 46.5%). Following a similar trend, more telehealth users reported having a comorbidity that they believed would make them more vulnerable to COVID-19 (448/898, 49.9%) than nonusers (405/1187, 31.4%). When non-telehealth users are split by veteran status, nonveterans report having no insurance in noticeably higher proportions: 11.5% (69/601) of nonveterans who did not use telehealth reported having no insurance compared to 0.9% (5/586) of veterans. More than half of telehealth users (604/898, 67.3%) and nonusers (736/1187, 62%) reported an income of US $50,000 or more. The median income of veteran and nonveteran groups was the same as the overall population, regardless of telehealth use.

The most common reason for using telehealth was for a regular check-up, followed by care for a prior health problem for veterans or for mental health care for nonveterans. Veterans who reported using telehealth for mental health care were slightly younger than veterans overall, with 10% (4/40) of mental health users falling at or younger than 44 years of age compared to 1.5% (9/586) of veterans who did not use telehealth. Similarly, a higher proportion of nonveterans who reported using telehealth for mental health care were 44 years or younger—72.1% (88/122) compared to 41.4% (247/601) of nonveterans who did not use telehealth. Among those who used telehealth, 525 (58.5%) reported much or somewhat preferring an in-person visit. Preference for in-person visits was higher among veterans, with 336 (70.9%) reporting that they much or somewhat preferred an in-person visit compared to 189 (44.6%) of nonveterans.

The most common reason for not using telehealth was “I haven’t needed to” (n=938 nonusers, 44.8%), followed by not feeling comfortable doing a telehealth visit (n=183 nonusers, 15.5%). See Table 2 for additional details on reasons for using and not using telehealth.

Most respondents felt that the difficulty of accessing health care was the same as before the pandemic (1203/2085, 57.7%), but 29.8% (622/2085) reported getting health care as being somewhat or much harder. Of the 367 respondents who reported a family member took part in a telehealth visit, 49 (13.3%) stated that their family member would not have been able to attend if the visit had been in person.

A larger proportion of veterans who used VA care reported having 2 or more comorbidities, whether as a mix or their primary source (62/110, 56.9% mixed and 104/169, 62.3% primarily VA vs 333/781, 43% for non-VA care). A higher proportion of veterans who primarily used VA care also reported greater difficulty in getting care since the beginning of the pandemic (84/169, 49.7% compared to 34/110, 30.9% for mixed care and 225/781, 28.8% for non-VA).

Veterans who used telehealth and had either mixed or primarily VA care had more total visits on average (mean 3.0, SD 2.7; mean 2.8, SD 2.2) than those who received care from non-VA sources (mean 2.1, SD 1.7), but all groups had the same median number of visits (median 2, IQR 1-3 for mixed or primarily VA care; median 2, IQR 1-2 for non-VA care). Those who used VA care reported more visits for mental health (10/65, 15.4% mixed care and 17/112, 15.2% primarily VA) than those who primarily used non-VA care (13/297, 4.4%). They also reported more visits for a general checkup (49/65, 75.4% mixed care and 71/112, 63.4% primarily VA) than those who used non-VA care (157/297, 52.9%).

**Table 1:** Characteristics of telehealth use.

			Nonveteran (n=424)	Veteran (n=474)	Total (N=898)
**Number of telehealth visits, n (%)**					
	1	157 (39.1)		183 (40)	340 (39.5)
	2	97 (24.1)		136 (29.7)	233 (27.1)
	3	52 (12.9)		52 (11.4)	104 (12.1)
	4	29 (7.2)		30 (6.6)	59 (6.9)
	5	16 (4.0)		16 (3.5)	32 (3.7)
	6	12 (3.0)		17 (3.7)	29 (3.4)
	7	5 (1.2)		6 (1.3)	11 (1.3)
	8	5 (1.2)		4 (0.9)	9 (1)
	9	4 (1.0)		1 (0.2)	5 (0.6)
	≥10	25 (6.2)		13 (2.8)	38 (4.4)
	Unknown	22 (5.2)		16 (3.4)	38 (4.2)
	Mean (SD)	2.8 (2.5)		2.5 (2.0)	2.6 (2.3)
**Prefer telehealth or in-person, n (%)**					
	Much prefer in person	117 (27.6)		239 (50.4)	356 (39.6)
	Somewhat prefer in person	72 (17.2)		97 (20.5)	169 (18.8)
	No preference	89 (21)		91 (19.2)	180 (20)
	Somewhat prefer telehealth	67 (15.8)		32 (6.8)	99 (11)
	Much prefer telehealth	79 (18.6)		15 (3.2)	94 (10.5)
**Have family members taken part in a telehealth visit? n (%)**					
	Unknown	3 (0.7)		0 (0)	3 (0.3)
	No	212 (50)		316 (66.7)	528 (58.8)
	Yes	209 (49.3)		158 (33.3)	367 (40.9)
**Would the family member or other loved one have been able to go to the visit if it had been in-person? n (%)**					
	Unknown	215 (50.7)		316 (66.7)	531 (59.1)
	Maybe	65 (15.3)		37 (7.8)	102 (11.4)
	No	27 (6.4)		22 (4.6)	49 (5.5)
	Yes	117 (27.6)		99 (20.9)	216 (24.1)

**Table 2:** Reasons for using or not using telehealth.

		Nonveteran		Veteran		Total
**Reasons for not using telehealth, n (%)^a^**						
	Overall	598	586		1184	
	I have not needed to	458 (76.6)	476 (81.2)		934 (78.9)	
	It was hard to get appointment	10 (1.7)	4 (0.7)		14 (1.2)	
	Provider does not offer telehealth	45 (7.5)	23 (3.9)		68 (5.7)	
	My insurance will not cover it	25 (4.2)	5 (0.9)		30 (2.5)	
	I do not have a smart phone/web cam	16 (2.7)	46 (7.8)		62 (5.2)	
	Internet is not good enough	11 (1.8)	4 (0.7)		15 (1.3)	
	Do not feel comfortable	91 (15.2)	92 (15.7)		183 (15.5)	
	Other	27 (4.5)	37 (6.3)		64 (5.4)	
**Reasons for using telehealth, n (%)^a^**						
	Overall	424	474		898	
	Mental health	122 (28.8)	40 (8.4)		162 (18.0)	
	Regular check-up	185 (43.6)	277 (58.4)		462 (51.4)	
	Prior health problem	105 (24.8)	178 (37.6)		283 (31.5)	
	New health problem	85 (20)	93 (19.6)		178 (19.8)	
	Thought I might have COVID-19	39 (9.2)	13 (2.7)		52 (5.8)	
	Child visit	36 (8.5)	4 (0.8)		40 (4.5)	
	Visit for someone else	21 (5.0)	15 (3.2)		36 (4.0)	
	Other	23 (5.4)	31 (6.5)		54 (6.0)	

^a^Answers were not mutually exclusive; percentages may add up to more than 100.

### Model Results

#### Likelihood of Using Telehealth (Zero-Inflated Model)

Within our population, Black respondents, those with 1 or more comorbidities, receiving health care equally at VA and non-VA facilities or primarily at the VA, having insurance, having a higher numeracy score, having “Unknown” education status, and having an income of US $50,000-US $99,999 or US $150,000+ were more likely to use telehealth (see Table S2 in Multimedia Appendix 1).

Respondents 45 years or older had a decreased likelihood of reporting telehealth use. For example, respondents aged 55-64 years were 0.39 times or 61% less likely to report a telehealth visit compared to respondents aged 18-34 years (95% CI 0.25-0.62). Midwest and southern regions were also associated with decreased usage of telehealth compared to the western regions. Living in a small city or suburban area was also associated with decreased usage of telehealth.

#### Number of Telehealth Visits Among Users

On average, being a veteran, receiving health care equally at VA and non-VA facilities or primarily at the VA, and preferring not to report income were associated with more telehealth visits. Respondents aged 55 years and older had fewer telehealth visits; on average, they reported 57% fewer visits (IRR 0.43, 95% CI 0.28-0.66) than those in the 18-34 years age category. Living in a rural location (IRR 0.66, 95% CI 0.48-0.92) or a small city (IRR 0.71, 95% CI 0.51-0.99) was also associated with fewer telehealth visits compared to living in a large city. Additionally, a higher numeracy score (our proxy for education) was associated with fewer telehealth visits, reversing the direction of association from the zero-inflated model (IRR 0.88, 95% CI 0.81-0.96). Education was not associated with the number of visits except for Trade school, which was associated with fewer telehealth visits (IRR 0.51, 95% CI 0.28-0.93).

## Discussion

Nearly half of the respondents surveyed reported using telehealth during the COVID-19 pandemic. Compared to nonveterans, veterans reported using telehealth more often, but most reported a preference for in-person visits. For both veterans and nonveterans, the most common reason for using telehealth was a regular check-up; the second most common reason differed between veterans (for prior health) and nonveterans (for mental health). We found that some groups, including those 45 years or older and those living in small cities, were less likely to use telehealth, while those who received primarily or mixed VA care were more likely to use it and had more visits when they did.

Our finding that increasing age is associated with less telehealth use is supported by previous literature [15,16]. An analysis of outpatient encounters in the VA by Ferguson et al [15] found that US veterans aged 45-65 years and older were less likely to use video care compared to younger age groups [15]. There are many potential reasons why older adults may be less likely to use telehealth, but the most commonly reported reason for not using telehealth in our study, after not having a need for it, was a lack of comfort with it. This would also be consistent with previous work, as a survey of rural older adults in 2021 found more than half of their sample would be unwilling to use telehealth for mental health services during the pandemic, and nearly half felt uncomfortable at the idea of using it for this purpose.

In all models, a higher number of comorbidities was associated with increased telehealth use but not the number of visits. The presence of multiple chronic conditions is associated with increased health care usage [14], which supports our finding of increased telehealth use, but the reason for the lack of association with the number of visits remains unclear. It is possible that the time period was too limited to see a difference in the number of visits, or that additional health care use beyond the first telehealth visit was performed in person.

The VA was an early adopter of telehealth and has a long history of initiatives to increase usage, especially among rural veterans [28]. They also are well established as providers of comprehensive mental health services and have integrated these services into primary care [29]. Given this, it was initially surprising that using telehealth for mental health services was more common among nonveterans. However, it is possible that individuals may have sought mental health services through in-person methods instead. This finding may also be due to differences between veterans and nonveterans in our sample. It should also be noted that most veterans within this sample reported receiving health care through non-VA sources, so any benefits derived from acquiring health care from the VA may not be as prominent in this group. For instance, veterans who reported receiving health care from mixed or primarily VA sources reported almost 4 times as much telehealth usage for mental health reasons as those using primarily non-VA health care. Nonetheless, this may provide guidance for the type of outreach needed to better care for these underserved groups. Future research on the types of health care most suitable and acceptable for delivery via telehealth is warranted.

Higher-income individuals were generally more likely to use telehealth when compared to respondents with incomes of <US $50,000 in this analysis, but only the “Prefer not to say” group had statistically significantly more visits. Prior literature has demonstrated a mixed relationship between income and telehealth use. An assessment of VA outpatient encounters found patients with low income to be more likely to receive web-based care during COVID-19, while a study from a non-VA facility found patients with low income to have lower odds of completing a web-based visit [15,17]. Some differences in telehealth use may be accounted for by the health care system used. For example, we found receiving health care primarily at the VA to be associated with more telehealth use and visits, but these veterans also had a lower median income than those who received a mix of VA care or non-VA care. This may suggest a difference between the people who seek out the VA for telehealth and those who use it outside of the VA system.

Several studies have reported patient satisfaction with telehealth to be high overall [19,30]. However, a majority (525/898, 58.5%) of respondents in our sample who used telehealth reported much or somewhat preferring in-person visits. It should be noted that this proportion is substantially different amongst nonveterans and veterans—44.6% (189/424) versus 70.9% (336/474) of telehealth users reported preferring in-person visits, respectively. This is likely due to underlying differences in our sample groups; for example, our veteran population is composed of individuals who are older and generally reported higher income than our nonveteran population.

There may be other reasons why individuals chose to use or not use telehealth. Hong et al [31] examined the relationship between internet search volume data for telehealth and existing hospital capacity for telemedicine. Their results found no correlation between interest in telehealth and the proportion of hospitals in the region offering telehealth services [31]. This may indicate a disconnect between the supply and demand of these services, as well as underuse in some areas. Other work suggests that individual factors influence telehealth use, including socioeconomic status, gender, and age [32-34]. As pandemic restrictions have eased, telehealth use has declined but remains above prepandemic levels [35]. Given the observed benefits of access to care, cost, and recent legislation supporting telehealth, this increased use will likely continue into the future [36-38].

This study has several potential limitations to consider. This study relies on self-reported data, which has the potential to be biased or inaccurate. However, while discrepancies have been observed between self-reported health care usage and electronic records, self-reported data have been shown to be predictive of medical records [39,40]. Future studies on telehealth could improve this method by supporting self-reported data with clinical data.

While there are many potential types of telehealth (eg, video chat, telephone, and text-based communication), all types are grouped together here. User experiences with different types of telemedicine may vary, as may the providers of each service.

Finally, our population contains a sample of veterans and nonveterans who were willing to take a web-based survey. As our selection process was not random, no matching was used, and this was an observational study, differences may be observed in these data, which may not reflect the underlying population.

This study also has several strengths. By using self-reported patient responses, we have the opportunity to discover patient experiences from their own perspective. While not random, our population contains over 2000 individuals from varying backgrounds and veteran status. In addition, this study includes telehealth encounters and reported health care use from both inside and outside of the VA, which can be missed when health care use is assessed within 1 facility or system.

We observed that individuals living in small cities and older adults are less likely to use telehealth and have fewer visits if they do use it. This suggests that, despite changes in health care systems and policies to support telehealth during the pandemic, some groups are still underserved. The examples of these policies include expansions in coverage for telehealth services, a broadening of allowed technology, and allowing for controlled substances to be prescribed through telehealth [41-43]. While some groups remain underserved, these efforts have produced results—in 1 striking example, US veterans who were provided with video-enabled tablets for telehealth use during the pandemic had a 36% reduction in suicide-related emergency department visits [44]. Telehealth has the potential to improve the lives of many people, but as we have demonstrated here, barriers to telehealth use still exist. However, these barriers are surmountable. Internet access can be improved, and in some cases, the distribution of telehealth-enabled devices can remove the barrier of not owning an appropriate device. Providers can be educated to better conduct telehealth services [45]. Concerns around comfort and privacy can be addressed through educational interventions [46]. Telehealth is an essential component of health care and has tremendous potential applications in the context of situations like COVID-19 [47,48]. Future research should address ways to reduce these barriers and improve telehealth experiences for both general practice and disaster-related use.

## Appendix

Multimedia Appendix 1 Demographics table, regression table, additional analyses, and survey questions used for this study.

## Abbreviations

IRR: incidence rate ratio
OR: odds ratio
VA: Veterans Health Administration
