# Supplementary material for: Telehealth Use During the COVID-19 Pandemic Among Veterans and Nonveterans: Web-Based Survey Study
Source: JMIR Form Res. 2023 Sep 8;7:e42217. doi: 10.2196/42217 (PMC10494868; doi:10.2196/42217)
Supplement: Multimedia Appendix 1 [file formative_v7i1e42217_app1.docx]

**Appendix**

**Title**: Telehealth use during the COVID-19 pandemic among Veterans and non-Veterans: An Online Survey

This appendix provides the main demographics and regression model results, results from additional analyses, and a table of the questions and available response scales used in the current study.

Table of Contents

[Main Paper Models 2](#_Toc142571138)

[Demographics 2](#_Toc142571139)

[Hurdle Model 6](#_Toc142571140)

[Veteran Status Stratified Models 9](#_Toc142571141)

[Veteran Only Model 9](#_Toc142571142)

[Non-Veteran Only Model 11](#_Toc142571143)

[Survey Questions and Response Scales 14](#_Toc142571144)

# Main Paper Models

## Demographics

**Table S1.** Demographics from a survey of veterans and nonveterans in December 2020, stratified by telehealth use.

|  | **Nonveteran n (%)** | | | **Veteran n (%)** | | |
| --- | --- | --- | --- | --- | --- | --- |
|  | **Telehealth Use**** | | | **Telehealth Use** | | |
|  | **No** | **Yes** | **All** | **No** | **Yes** | **All** |
| **Overall** | 598 (100%) | 424 (100%) | 1025 (100%) | 586 (100%) | 474 (100%) | 1060 (100%) |
| **Age** |  |  |  |  |  |  |
| Unknown | 1 | 4 | 5 (0.5%) | 1 | 1 | 2 (0.2%) |
| 18-34 | 135 (22.6%) | 129 (30.7%) | 265 (26.0%) | 1 (0.2%) | 5 (1.1%) | 6 (0.6%) |
| 35 - 44 | 111 (18.6%) | 96 (22.9%) | 207 (20.3%) | 8 (1.4%) | 5 (1.1%) | 13 (1.2%) |
| 45 - 54 | 80 (13.4%) | 52 (12.4%) | 134 (13.1%) | 10 (1.7%) | 16 (3.4%) | 26 (2.5%) |
| 55 - 64 | 67 (11.2%) | 35 (8.3%) | 102 (10.0%) | 67 (11.5%) | 67 (14.2%) | 134 (12.7%) |
| 65 - 74 | 164 (27.5%) | 87 (20.7%) | 251 (24.6%) | 313 (53.5%) | 249 (52.6%) | 562 (53.1%) |
| 75 - 84 | 35 (5.9%) | 21 (5.0%) | 56 (5.5%) | 168 (28.7%) | 114 (24.1%) | 282 (26.7%) |
| 85 or older | 5 (0.8%) | 0 (0.0%) | 5 (0.5%) | 18 (3.1%) | 17 (3.6%) | 35 (3.3%) |
| **Gender** |  |  |  |  |  |  |
| Any Other | 10 (1.7%) | 7 (1.7%) | 17 (1.7%) | 1 (0.2%) | 0 (0.0%) | 1 (0.1%) |
| Female | 324 (54.2%) | 226 (53.3%) | 551 (53.8%) | 44 (7.5%) | 40 (8.4%) | 84 (7.9%) |
| Male | 264 (44.1%) | 191 (45.0%) | 457 (44.6%) | 541 (92.3%) | 434 (91.6%) | 975 (92.0%) |
| **Race*** |  |  |  |  |  |  |
| American Indian or Alaskan Native | 5 (0.8%) | 3 (0.7%) | 8 (0.8%) | 8 (1.4%) | 3 (0.6%) | 11 (1.0%) |
| Asian or Asian American | 24 (4.0%) | 16 (3.8%) | 40 (3.9%) | 9 (1.5%) | 13 (2.7%) | 22 (2.1%) |
| Black or African American | 68 (11.4%) | 64 (15.1%) | 133 (13.0%) | 56 (9.6%) | 73 (15.4%) | 129 (12.2%) |
| Native Hawaiian or other Pacific Islander | 1 (0.2%) | 0 (0.0%) | 1 (0.1%) | 3 (0.5%) | 2 (0.4%) | 5 (0.5%) |
| White or European American | 500 (83.6%) | 340 (80.2%) | 842 (82.1%) | 503 (85.8%) | 376 (79.3%) | 879 (82.9%) |
| Any Other | 5 (0.8%) | 3 (0.7%) | 7 ( 0.7%) | 17 (2.9%) | 18 (3.8%) | 30 (2.8%) |
| **Numeracy Score** |  |  |  |  |  |  |
| Mean (SD) | 4.4 (1.4) | 4.5 (1.3) | 4.4 (1.4) | 5.0 (1.1) | 5.0 (1.1) | 5.0 (1.1) |
| **Education** |  |  |  |  |  |  |
| Unknown | 338 | 297 | 638 | 0 | 0 | 0 |
| High School or less | 38 (14.6%) | 18 (14.2%) | 56 (14.5%) | 65 (11.1%) | 38 (8.0%) | 103 (9.7%) |
| Some College | 60 (23.1%) | 29 (22.8%) | 89 (23.0%) | 138 (23.5%) | 112 (23.6%) | 250 (23.6%) |
| Trade School | 7 (2.7%) | 3 (2.4%) | 10 (2.6%) | 48 (8.2%) | 38 (8.0%) | 86 (8.1%) |
| Bachelor's Degree | 85 (32.7%) | 46 (36.2%) | 131 (33.9%) | 200 (34.1%) | 173 (36.5%) | 373 (35.2%) |
| Master’s degree | 48 (18.5%) | 29 (22.8%) | 77 (19.9%) | 102 (17.4%) | 90 (19.0%) | 192 (18.1%) |
| Doctoral/Professional degree | 22 (8.5%) | 2 (1.6%) | 24 (6.2%) | 33 (5.6%) | 23 (4.9%) | 56 (5.3%) |
| **Income** |  |  |  |  |  |  |
| Less than $20,000 | 87 (14.5%) | 63 (14.9%) | 151 (14.7%) | 21 (3.6%) | 17 (3.6%) | 38 (3.6%) |
| $20,000 - $29,999 | 51 (8.5%) | 29 (6.8%) | 81 (7.9%) | 25 (4.3%) | 21 (4.4%) | 46 (4.3%) |
| $30,000 - $39,999 | 40 (6.7%) | 32 (7.5%) | 72 (7.0%) | 44 (7.5%) | 26 (5.5%) | 70 (6.6%) |
| $40,000 - $49,999 | 53 (8.9%) | 29 (6.8%) | 82 (8.0%) | 58 (9.9%) | 41 (8.6%) | 99 (9.3%) |
| $50,000 - $59,999 | 39 (6.5%) | 39 (9.2%) | 78 (7.6%) | 44 (7.5%) | 43 (9.1%) | 87 (8.2%) |
| $60,000 - $74,999 | 57 (9.5%) | 55 (13.0%) | 112 (10.9%) | 80 (13.7%) | 59 (12.4%) | 139 (13.1%) |
| $75,000 - $99,999 | 74 (12.4%) | 53 (12.5%) | 127 (12.4%) | 97 (16.6%) | 90 (19.0%) | 187 (17.6%) |
| $100,000 - $149,999 | 95 (15.9%) | 53 (12.5%) | 149 (14.5%) | 113 (19.3%) | 97 (20.5%) | 210 (19.8%) |
| $150,000+ | 60 (10.0%) | 51 (12.0%) | 111 (10.8%) | 76 (13.0%) | 64 (13.5%) | 140 (13.2%) |
| Prefer to not say | 42 (7.0%) | 20 (4.7%) | 62 (6.0%) | 28 (4.8%) | 16 (3.4%) | 44 (4.2%) |
| **US Region** |  |  |  |  |  |  |
| West | 93 (15.6%) | 75 (17.7%) | 168 (16.4%) | 122 (20.8%) | 124 (26.2%) | 246 (23.2%) |
| Midwest | 145 (24.2%) | 85 (20.0%) | 230 (22.4%) | 128 (21.8%) | 73 (15.4%) | 201 (19.0%) |
| Northeast | 122 (20.4%) | 107 (25.2%) | 230 (22.4%) | 93 (15.9%) | 77 (16.2%) | 170 (16.0%) |
| South | 238 (39.8%) | 151 (35.6%) | 390 (38.0%) | 242 (41.3%) | 195 (41.1%) | 437 (41.2%) |
| Other | 0 (0.0%) | 6 (1.4%) | 7 (0.7%) | 1 (0.2%) | 5 (1.1%) | 6 (0.6%) |
| **Location Type** |  |  |  |  |  |  |
| Unknown | 0 | 3 | 3 (0.2%) | 0 | 0 | 0 |
| Large city, more than 1 million | 65 (10.9%) | 80 (19.0%) | 145 (14.2%) | 46 (7.8%) | 42 (8.9%) | 88 (8.3%) |
| Mid-sized city, 100,000 to 1 million people | 57 (9.5%) | 36 (8.6%) | 94 (9.2%) | 54 (9.2%) | 48 (10.1%) | 102 (9.6%) |
| Other, please specify | 1 (0.2%) | 1 (0.2%) | 2 (0.2%) | 1 (0.2%) | 4 (0.8%) | 5 (0.5%) |
| Rural | 121 (20.2%) | 86 (20.4%) | 207 (20.3%) | 106 (18.1%) | 79 (16.7%) | 185 (17.5%) |
| Small city, e.g. less than 100,000 people | 99 (16.6%) | 63 (15.0%) | 162 (15.9%) | 114 (19.5%) | 83 (17.5%) | 197 (18.6%) |
| Suburban, near a Large city | 255 (42.6%) | 155 (36.8%) | 412 (40.3%) | 265 (45.2%) | 218 (46.0%) | 483 (45.6%) |
| **Number of Comorbidities^+^** |  |  |  |  |  |  |
| Unknown | 5 | 7 | 12 (1.2%) | 6 | 3 | 9 (0.8%) |
| None | 301 (50.8%) | 124 (29.7%) | 427 (42.2) | 179 (30.9%) | 70 (14.9%) | 249 ( 23.7) |
| One | 134 (22.6%) | 99 (23.7%) | 233 (23.0) | 191 (32.9%) | 112 (23.8%) | 303 ( 28.8) |
| Two or more | 158 (26.6%) | 194 (46.5%) | 353 (34.8) | 210 (36.2%) | 289 (61.4%) | 499 ( 47.5) |
| **COVID Risk Condition^++^** |  |  |  |  |  |  |
| Yes | 167 (27.9%) | 167 (39.4%) | 336 (32.8%) | 236 (40.3%) | 281 (59.3%) | 517 (48.8%) |
| No | 386 (64.5%) | 224 (52.8%) | 611 (59.6%) | 298 (50.9%) | 150 (31.6%) | 448 (42.3%) |
| Unsure | 40 (6.7%) | 32 (7.5%) | 72 (7.0%) | 51 (8.7%) | 43 (9.1%) | 94 (8.9%) |
| Unknown | 5 (0.8%) | 1 (0.2%) | 6 (0.6%) | 1 (0.2%) | 0 (0.0%) | 1 (0.1%) |
| **Job change due to COVID** |  |  |  |  |  |  |
| Yes | 118 (19.8%) | 121 (28.5%) | 240 (23.4%) | 54 (9.2%) | 59 (12.5%) | 113 (10.7%) |
| No | 479 (80.2%) | 303 (71.5%) | 784 (76.6%) | 532 (90.8%) | 413 (87.5%) | 945 (89.3%) |
| Unknown | 1 | 0 | 1 (0.1%) | 0 | 2 | 2 (0.2%) |
| **Primary Health care Source** |  |  |  |  |  |  |
| Non-VA | 598 (100.0%) | 424 (100.0%) | 1025 (100.0%) | 484 (82.6%) | 297 (62.7%) | 781 (73.7%) |
| About an equal mix of VA and non VA | 0 (0.0%) | 0 (0.0%) | 0 (0.0%) | 45 (7.7%) | 65 (13.7%) | 110 (10.4%) |
| Primarily at VA | 0 (0.0%) | 0 (0.0%) | 0 (0.0%) | 57 (9.7%) | 112 (23.6%) | 169 (15.9%) |
| **Insurance** |  |  |  |  |  |  |
| None | 69 (11.5%) | 28 (6.6%) | 97 (9.5%) | 5 (0.9%) | 5 (1.1%) | 10 (0.9%) |
| Any | 529 (88.5%) | 396 (93.4%) | 928 (90.5%) | 581 (99.1%) | 469 (98.9%) | 1050 (99.1%) |
| **Access to Health care since pandemic began** |  |  |  |  |  |  |
| Unknown | 2 | 2 | 4 | 0 | 1 | 1 |
| Much harder to get health care | 49 (8.2%) | 31 (7.3%) | 80 (7.8%) | 22 (3.8%) | 41 (8.7%) | 63 (5.9%) |
| Somewhat harder to get health care | 103 (17.3%) | 95 (22.5%) | 199 (19.4%) | 131 (22.4%) | 149 (31.5%) | 280 (26.4%) |
| Same as before COVID | 349 (58.6%) | 205 (48.6%) | 555 (54.1%) | 398 (67.9%) | 250 (52.9%) | 648 (61.1%) |
| Somewhat easier to get health care | 71 (11.9%) | 44 (10.4%) | 115 (11.2%) | 33 (5.6%) | 21 (4.4%) | 54 (5.1%) |
| Much easier to get health care | 24 (4.0%) | 47 (11.1%) | 72 (7.0%) | 2 (0.3%) | 12 (2.5%) | 14 (1.3%) |
| *Answers were not mutually exclusive; percentages may add up to more than 100 | | | | | | |
| **Three respondents had missing telehealth data | | | | | | |
| ^+^Listed comorbidities included Asthma, emphysema, or chronic bronchitis, COPD (other lung disease); Arthritis or rheumatism; Cancer, diagnosed in the past 3 years; Diabetes; Digestive problems (such as ulcer, colitis, or gallbladder disease); Heart trouble (such as angina, congestive heart failure, or coronary artery disease, having a past heart attack); HIV illness or AIDS; Kidney disease; Liver problems (such as cirrhosis); Stroke; High blood pressure (hypertension); Very overweight or obese | | | | | | |
| ^++^Assessed through the question, “Do you have a preexisting health condition—respiratory illness, cancer, heart disease, high blood pressure, etc.—that you believe makes you more vulnerable to coronavirus?” | | | | | | |

## Hurdle Model

**Table S2.** Incidence rate ratios and odds ratios from a 2-part hurdle model assessing likelihood of telehealth use, and number of visits among users.

|  | **Telehealth Visits Two-Part model, Wave 1** | | | | | |
| --- | --- | --- | --- | --- | --- | --- |
|  | **Number of telehealth visits among users (Count Model)** | | | **Likelihood of using telehealth (Zero-Inflated Model)** | | |
| *Predictors* | *Incidence Rate Ratios* | *CI* | *P* | *Odds Ratios* | *CI* | *P* |
| (Intercept) | 3.51 | 1.06 – 11.58 | **.04** | 0.08 | 0.02 – 0.28 | **<.001** |
| **Veteran status** |  |  |  |  |  |  |
| Non-Veteran |  | Ref |  |  | Ref |  |
| Veteran | 1.49 | 1.07 – 2.07 | **.02** | 1.33 | 0.97 – 1.82 | .08 |
| **Age** |  |  |  |  |  |  |
| 18 – 34 |  | Ref |  |  | Ref |  |
| 35 – 44 | 0.86 | 0.62 – 1.21 | .40 | 0.68 | 0.45 – 1.02 | .06 |
| 45 – 54 | 1.07 | 0.74 – 1.55 | .73 | 0.53 | 0.34 – 0.83 | **.006** |
| 55 – 64 | 0.43 | 0.28 – 0.66 | **<.001** | 0.39 | 0.25 – 0.62 | **<.001** |
| 65 – 74 | 0.49 | 0.34 – 0.71 | **<.001** | 0.35 | 0.23 – 0.53 | **<.001** |
| 75 – 84 | 0.54 | 0.35 – 0.82 | **.004** | 0.3 | 0.19 – 0.48 | **<.001** |
| 85 or older | 0.44 | 0.21 – 0.93 | **.03** | 0.33 | 0.15 – 0.73 | **.006** |
| **Gender** |  |  |  |  |  |  |
| Male |  | Ref |  |  | Ref |  |
| Any Other | 1.27 | 0.49 – 3.32 | .62 | 0.84 | 0.29 – 2.44 | .75 |
| Female | 1.27 | 0.99 – 1.61 | .06 | 1.04 | 0.80 – 1.36 | .76 |
| **Race** |  |  |  |  |  |  |
| American Indian or Alaskan Native | 1.81 | 0.65 – 5.03 | .25 | 0.66 | 0.22 – 2.03 | .47 |
| Asian or Asian American | 1.33 | 0.52 – 3.39 | .55 | 2.59 | 0.92 – 7.27 | .07 |
| Black or African American | 1.22 | 0.49 – 3.05 | .67 | 2.87 | 1.04 – 7.90 | **.04** |
| Native Hawaiian or other Pacific Islander | 0.63 | 0.07 – 5.92 | .68 | 0.17 | 0.02 – 1.31 | .09 |
| White or European American | 1.31 | 0.54 – 3.19 | .55 | 2.14 | 0.80 – 5.73 | .13 |
| Any Other | 2.1 | 0.73 – 6.03 | .17 | 2.54 | 0.77 – 8.42 | .13 |
| **US Region** |  |  |  |  |  |  |
| West |  | Ref |  |  | Ref |  |
| Midwest | 0.89 | 0.66 – 1.19 | .44 | 0.6 | 0.44 – 0.82 | **.001** |
| Northeast | 1.06 | 0.81 – 1.41 | .66 | 0.91 | 0.67 – 1.24 | .54 |
| South | 0.94 | 0.73 – 1.20 | .60 | 0.69 | 0.53 – 0.91 | **.008** |
| US Territory or Other | 1.98 | 0.80 – 4.86 | .14 | 5.55 | 0.57 – 53.74 | .14 |
| **Location Type** |  |  |  |  |  |  |
| Large City, more than 1 million |  | Ref |  |  | Ref |  |
| Mid-sized city, 100,000 to 1 million people | 0.75 | 0.51 – 1.08 | .13 | 0.66 | 0.43 – 1.02 | .06 |
| Suburban, near a large city | 0.78 | 0.59 – 1.03 | .09 | 0.71 | 0.51 – 0.98 | **.040** |
| Small city, e.g. less than 100,000 people | 0.71 | 0.51 – 0.99 | **.04** | 0.63 | 0.43 – 0.92 | **.018** |
| Rural | 0.66 | 0.48 – 0.92 | **.01** | 0.7 | 0.48 – 1.02 | .06 |
| Other | 0.43 | 0.12 – 1.54 | .19 | 3.87 | 0.39 – 38.97 | .25 |
| **Number of Comorbidities** |  |  |  |  |  |  |
| 0 |  | Ref |  |  | Ref |  |
| 1 | 0.89 | 0.67 – 1.17 | .41 | 2.03 | 1.55 – 2.66 | **<.001** |
| 2 or more | 1.23 | 0.96 – 1.57 | .10 | 4.13 | 3.21 – 5.31 | **<.001** |
| **Job change due to COVID** |  |  |  |  |  |  |
| No |  | Ref |  |  | Ref |  |
| Yes | 1.05 | 0.84 – 1.33 | .66 | 1.23 | 0.94 – 1.60 | .14 |
| **Primary Healthcare Source** |  |  |  |  |  |  |
| Non-VA^a^ |  | Ref |  |  | Ref |  |
| About an equal mix of VA and non VA | 1.57 | 1.11 – 2.24 | **.01** | 2.18 | 1.40 – 3.39 | **.001** |
| Primarily at VA | 1.5 | 1.10 – 2.04 | **.01** | 3.25 | 2.20 – 4.81 | **<.001** |
| **Insurance** |  |  |  |  |  |  |
| None |  | Ref |  |  | Ref |  |
| Any | 0.85 | 0.53 – 1.38 | .52 | 2.1 | 1.26 – 3.49 | **.004** |
| **Income** |  |  |  |  |  |  |
| < $50,000 |  | Ref |  |  | Ref |  |
| $50,000 - $59,999 | 0.8 | 0.56 – 1.14 | .22 | 1.62 | 1.10 – 2.40 | **.02** |
| $60,000 - $74,999 | 1.07 | 0.78 – 1.45 | .69 | 1.51 | 1.07 – 2.12 | **.02** |
| $75,000 - $99,999 | 1 | 0.73 – 1.35 | .97 | 1.4 | 1.01 – 1.94 | **.04** |
| $100,000 - $149,999 | 1.21 | 0.88 – 1.66 | .24 | 1.24 | 0.89 – 1.72 | .20 |
| $150,000+ | 1.23 | 0.87 – 1.73 | .24 | 1.55 | 1.07 – 2.24 | **.02** |
| Prefer to not say | 1.72 | 1.07 – 2.77 | **.03** | 1.2 | 0.74 – 1.95 | .47 |
| **Numeracy** | 0.88 | 0.81 – 0.96 | **.002** | 1.1 | 1.00 – 1.20 | **.04** |
| **Education** |  |  |  |  |  |  |
| High School or Less |  | Ref |  |  | Ref |  |
| Unknown | 1.07 | 0.68 – 1.67 | .78 | 1.77 | 1.12 – 2.80 | **.01** |
| Some College | 0.82 | 0.54 – 1.24 | .34 | 1.19 | 0.76 – 1.85 | .44 |
| Trade School | 0.51 | 0.28 – 0.93 | **.03** | 0.94 | 0.52 – 1.71 | .84 |
| Bachelor's Degree | 0.81 | 0.54 – 1.21 | .30 | 1.29 | 0.84 – 1.98 | .25 |
| Master’s degree | 0.78 | 0.49 – 1.23 | .28 | 1.39 | 0.87 – 2.23 | .17 |
| Doctoral/Professional degree | 1.14 | 0.59 – 2.21 | .69 | 0.82 | 0.43 – 1.57 | .55 |
| Observations | 2013 | | |  |  |  |
| R^2^ / R^2^ adjusted | 0.371 / 0.357 | | |  |  |  |

^a^VA: Veterans Health Administration.

# Veteran Status Stratified Models

## Veteran Only Model

Table S3. Incidence rate ratios and odds ratios from a two-part hurdle model assessing likelihood of telehealth use, and number of visits among users. Population limited to veterans only. The “any other” gender category was dropped in this model due to low numbers.

|  | **Telehealth Visits Two-Part model, Wave 1** | | | | | |
| --- | --- | --- | --- | --- | --- | --- |
|  | **Number of telehealth visits among users (Count Model)** | | | **Likelihood of using telehealth (Zero-Inflated Model)** | | |
| *Predictors* | *Incidence Rate Ratios* | *CI* | *P* | *Odds Ratios* | *CI* | *P* |
| (Intercept) | 23.05 | 3.09 – 172.02 | **0.002** | 1.3 | 0.06 – 28.77 | 0.869 |
| **Age** |  |  |  |  |  |  |
| 18 – 34 |  | Ref |  |  | Ref |  |
| 35 – 44 | 0.69 | 0.19 – 2.56 | 0.580 | 0.05 | 0.00 – 0.67 | **0.025** |
| 45 – 54 | 0.83 | 0.30 – 2.32 | 0.721 | 0.09 | 0.01 – 1.11 | 0.06 |
| 55 – 64 | 0.35 | 0.14 – 0.92 | **0.033** | 0.08 | 0.01 – 0.81 | **0.033** |
| 65 – 74 | 0.34 | 0.14 – 0.84 | **0.020** | 0.07 | 0.01 – 0.73 | **0.026** |
| 75 – 84 | 0.41 | 0.16 – 1.05 | 0.063 | 0.05 | 0.01 – 0.58 | **0.016** |
| 85 or older | 0.29 | 0.10 – 0.85 | **0.025** | 0.08 | 0.01 – 0.90 | **0.041** |
| **Gender** |  |  |  |  |  |  |
| Male |  | Ref |  |  | Ref |  |
| Female | 1.01 | 0.66 – 1.54 | 0.98 | 0.93 | 0.54 – 1.60 | 0.794 |
| **Race** |  |  |  |  |  |  |
| American Indian or Alaskan Native | 0.3 | 0.03 – 2.84 | 0.293 | 0.37 | 0.08 – 1.71 | 0.204 |
| Asian or Asian American | 0.92 | 0.19 – 4.44 | 0.919 | 3.97 | 0.87 – 18.13 | 0.075 |
| Black or African American | 1.05 | 0.27 – 4.05 | 0.942 | 3.98 | 1.02 – 15.57 | **0.047** |
| Native Hawaiian or other Pacific Islander | 0.97 | 0.06 – 14.45 | 0.982 | 0.19 | 0.02 – 1.92 | 0.158 |
| White or European American | 0.97 | 0.25 – 3.80 | 0.966 | 2.73 | 0.72 – 10.41 | 0.141 |
| Any Other | 1.75 | 0.40 – 7.58 | 0.454 | 3.45 | 0.74 – 15.97 | 0.114 |
| **US Region** |  |  |  |  |  |  |
| West |  | Ref |  |  | Ref |  |
| Midwest | 0.99 | 0.68 – 1.43 | 0.951 | 0.56 | 0.36 – 0.86 | **0.008** |
| Northeast | 1.1 | 0.76 – 1.59 | 0.604 | 0.8 | 0.51 – 1.26 | 0.342 |
| South | 0.83 | 0.62 – 1.12 | 0.226 | 0.76 | 0.53 – 1.09 | 0.133 |
| US Territory or Other | 1.77 | 0.62 – 5.07 | 0.288 | 1.91 | 0.18 – 20.63 | 0.595 |
| **Location Type** |  |  |  |  |  |  |
| Large City, more than 1 million |  | Ref |  |  | Ref |  |
| Mid-sized city, 100,000 to 1 million people | 1.04 | 0.61 – 1.75 | 0.898 | 0.98 | 0.51 – 1.89 | 0.961 |
| Suburban, near a large city | 0.9 | 0.59 – 1.37 | 0.623 | 0.85 | 0.50 – 1.45 | 0.548 |
| Small city, e.g. less than 100,000 people | 0.91 | 0.56 – 1.47 | 0.696 | 0.79 | 0.44 – 1.42 | 0.428 |
| Rural | 0.94 | 0.58 – 1.53 | 0.808 | 0.84 | 0.46 – 1.52 | 0.566 |
| Other | 0.14 | 0.02 – 1.33 | 0.087 | 5.21 | 0.43 – 63.12 | 0.195 |
| **Number of Comorbidities** |  |  |  |  |  |  |
| 0 |  | Ref |  |  | Ref |  |
| 1 | 0.91 | 0.61 – 1.37 | 0.659 | 1.63 | 1.10 – 2.43 | **0.015** |
| 2 or more | 1.35 | 0.94 – 1.93 | 0.1 | 3.87 | 2.68 – 5.59 | **<0.001** |
| **Job change due to COVID** |  |  |  |  |  |  |
| No |  | Ref |  |  | Ref |  |
| Yes | 1.08 | 0.77 – 1.52 | 0.655 | 1.3 | 0.83 – 2.04 | 0.253 |
| **Primary Healthcare Source** |  |  |  |  |  |  |
| Non-VA |  | Ref |  |  | Ref |  |
| About an equal mix of VA and non VA | 1.54 | 1.11 – 2.15 | **0.01** | 2.28 | 1.45 – 3.57 | **<0.001** |
| Primarily at VA | 1.33 | 0.99 – 1.80 | 0.061 | 3.3 | 2.19 – 4.98 | **<0.001** |
| **Insurance** |  |  |  |  |  |  |
| None |  | Ref |  |  | Ref |  |
| Any | 0.44 | 0.17 – 1.13 | 0.089 | 0.58 | 0.14 – 2.45 | 0.455 |
| **Income** |  |  |  |  |  |  |
| < $50,000 |  | Ref |  |  | Ref |  |
| $50,000 - $59,999 | 0.85 | 0.52 – 1.38 | 0.500 | 1.55 | 0.89 – 2.72 | 0.121 |
| $60,000 - $74,999 | 0.99 | 0.66 – 1.48 | 0.952 | 1.39 | 0.85 – 2.26 | 0.188 |
| $75,000 - $99,999 | 1.11 | 0.75 – 1.64 | 0.595 | 1.67 | 1.06 – 2.64 | **0.027** |
| $100,000 - $149,999 | 1.22 | 0.83 – 1.81 | 0.311 | 1.68 | 1.06 – 2.66 | **0.028** |
| $150,000+ | 1.15 | 0.72 – 1.83 | 0.570 | 1.65 | 0.98 – 2.78 | 0.059 |
| Prefer to not say | 1.42 | 0.68 – 2.95 | 0.354 | 1.42 | 0.66 – 3.09 | 0.372 |
| **Numeracy** | 0.83 | 0.74 – 0.93 | **0.002** | 1.06 | 0.92 – 1.22 | 0.442 |
| **Education** |  |  |  |  |  |  |
| High School or Less |  | Ref |  |  | Ref |  |
| Some College | 0.77 | 0.48 – 1.23 | 0.275 | 1.38 | 0.80 – 2.38 | 0.254 |
| Trade School | 0.49 | 0.26 – 0.91 | **0.025** | 1.16 | 0.59 – 2.28 | 0.669 |
| Bachelor's Degree | 0.81 | 0.51 – 1.27 | 0.356 | 1.51 | 0.88 – 2.59 | 0.131 |
| Master’s degree | 0.77 | 0.46 – 1.29 | 0.318 | 1.49 | 0.82 – 2.70 | 0.186 |
| Doctoral/Professional degree | 0.9 | 0.45 – 1.83 | 0.779 | 1.48 | 0.68 – 3.22 | 0.324 |
| Observations | 1031 | | |  |  |  |
| R^2^ / R^2^ adjusted | 0.380 / 0.354 | | |  |  |  |

## Non-Veteran Only Model

Table S4. Incidence rate ratios and odds ratios from a two-part hurdle model assessing likelihood of telehealth use, and number of visits among users. Population limited to non-Veterans only. Due to low numbers, the 75-84 and 85 or older categories were combined, and the Native Hawaiian or other Pacific Islander race was omitted from the model. Additionally, the “Other” categories for the US Region and Location Type variables were combined with “West” and “Large city” categories, respectively (each “Other” category had 5 or less people and caused problems with the model when left alone).

|  | **Telehealth Visits Two-Part model, Wave 1** | | | | | |
| --- | --- | --- | --- | --- | --- | --- |
|  | **Number of telehealth visits among users (Count Model)** | | | **Likelihood of using telehealth (Zero-Inflated Model)** | | |
| *Predictors* | *Incidence Rate Ratios* | *CI* | *P* | *Odds Ratios* | *CI* | *P* |
| (Intercept) | 1.11 | 0.17 – 7.23 | 0.917 | 0.12 | 0.02 – 0.90 | **0.039** |
| **Age** |  |  |  |  |  |  |
| 18 – 34 |  | Ref |  |  | Ref |  |
| 35 – 44 | 0.88 | 0.61 – 1.26 | 0.476 | 0.74 | 0.49 – 1.14 | 0.174 |
| 45 – 54 | 1.08 | 0.71 – 1.64 | 0.724 | 0.54 | 0.33 – 0.88 | **0.013** |
| 55 – 64 | 0.36 | 0.19 – 0.67 | **0.002** | 0.36 | 0.20 – 0.65 | **0.001** |
| 65 – 74 | 0.55 | 0.34 – 0.88 | **0.014** | 0.31 | 0.19 – 0.50 | **<0.001** |
| 75 or older | 0.35 | 0.16 – 0.74 | **0.006** | 0.3 | 0.15 – 0.60 | **0.001** |
| **Gender** |  |  |  |  |  |  |
| Male |  | Ref |  |  | Ref |  |
| Any Other | 1.37 | 0.50 – 3.78 | 0.538 | 0.83 | 0.27 – 2.60 | 0.750 |
| Female | 1.54 | 1.11 – 2.13 | **0.009** | 1.06 | 0.77 – 1.46 | 0.707 |
| **Race** |  |  |  |  |  |  |
| American Indian or Alaskan Native | 7.29 | 1.51 – 35.12 | **0.013** | 1.09 | 0.15 – 7.80 | 0.929 |
| Asian or Asian American | 1.96 | 0.46 – 8.30 | 0.36 | 1.74 | 0.34 – 8.85 | 0.507 |
| Black or African American | 1.96 | 0.40 – 9.51 | 0.404 | 1.88 | 0.32 – 10.96 | 0.485 |
| White or European American | 2.62 | 0.58 – 11.88 | 0.211 | 1.55 | 0.28 – 8.58 | 0.618 |
| Any Other | 0.77 | 0.04 – 13.29 | 0.855 | 1.18 | 0.13 – 10.74 | 0.884 |
| **US Region** |  |  |  |  |  |  |
| West, US Territory, or Other |  | Ref |  |  | Ref |  |
| Midwest | 0.84 | 0.54 – 1.30 | 0.442 | 0.62 | 0.39 – 0.99 | **0.045** |
| Northeast | 1.01 | 0.67 – 1.52 | 0.957 | 0.98 | 0.62 – 1.54 | 0.925 |
| South | 1.03 | 0.70 – 1.53 | 0.874 | 0.58 | 0.38 – 0.88 | **0.011** |
| **Location Type** |  |  |  |  |  |  |
| Large City, more than 1 million, or other |  | Ref |  |  | Ref |  |
| Mid-sized city, 100,000 to 1 million people | 0.65 | 0.38 – 1.11 | 0.115 | 0.46 | 0.25 – 0.85 | **0.013** |
| Suburban, near a large city | 0.76 | 0.52 – 1.11 | 0.16 | 0.62 | 0.40 – 0.97 | **0.036** |
| Small city, e.g. less than 100,000 people | 0.64 | 0.39 – 1.03 | 0.068 | 0.54 | 0.32 – 0.92 | **0.024** |
| Rural | 0.5 | 0.32 – 0.78 | **0.002** | 0.62 | 0.37 – 1.03 | 0.065 |
| **Number of Comorbidities** |  |  |  |  |  |  |
| 0 |  | Ref |  |  | Ref |  |
| 1 | 0.8 | 0.54 – 1.19 | 0.269 | 2.56 | 1.74 – 3.75 | **<0.001** |
| 2 or more | 1.17 | 0.83 – 1.63 | 0.37 | 4.42 | 3.10 – 6.31 | **<0.001** |
| **Job change due to COVID** |  |  |  |  |  |  |
| No |  | Ref |  |  | Ref |  |
| Yes | 1.11 | 0.82 – 1.52 | 0.494 | 1.24 | 0.88 – 1.75 | 0.221 |
| **Insurance** |  |  |  |  |  |  |
| None |  | Ref |  |  | Ref |  |
| Any | 0.98 | 0.56 – 1.72 | 0.957 | 2.55 | 1.46 – 4.47 | **0.001** |
| **Income** |  |  |  |  |  |  |
| < $50,000 |  | Ref |  |  | Ref |  |
| $50,000 - $59,999 | 0.85 | 0.52 – 1.41 | 0.542 | 1.81 | 1.02 – 3.19 | **0.042** |
| $60,000 - $74,999 | 1.12 | 0.71 – 1.77 | 0.622 | 1.74 | 1.05 – 2.87 | **0.031** |
| $75,000 - $99,999 | 0.81 | 0.50 – 1.30 | 0.385 | 1.16 | 0.71 – 1.90 | 0.564 |
| $100,000 - $149,999 | 1.01 | 0.60 – 1.68 | 0.982 | 0.86 | 0.52 – 1.43 | 0.571 |
| $150,000+ | 1.4 | 0.86 – 2.28 | 0.181 | 1.56 | 0.90 – 2.70 | 0.109 |
| Prefer to not say | 1.99 | 1.04 – 3.82 | **0.039** | 1.12 | 0.59 – 2.13 | 0.739 |
| **Numeracy** | 0.94 | 0.84 – 1.06 | 0.319 | 1.15 | 1.01 – 1.30 | **0.031** |
| **Education** |  |  |  |  |  |  |
| High School or Less |  | Ref |  |  | Ref |  |
| Unknown | 1.12 | 0.57 – 2.20 | 0.738 | 1.28 | 0.67 – 2.44 | 0.452 |
| Some College | 0.85 | 0.38 – 1.94 | 0.705 | 0.89 | 0.41 – 1.95 | 0.773 |
| Trade School | 0.28 | 0.02 – 3.63 | 0.331 | 0.6 | 0.10 – 3.58 | 0.573 |
| Bachelor's Degree | 0.94 | 0.42 – 2.11 | 0.886 | 0.96 | 0.45 – 2.03 | 0.910 |
| Master’s degree | 0.85 | 0.35 – 2.08 | 0.728 | 1.18 | 0.52 – 2.65 | 0.697 |
| Doctoral/Professional degree | 4.6 | 0.84 – 25.04 | 0.078 | 0.14 | 0.03 – 0.74 | **0.020** |
| Observations | 981 | | |  |  |  |
| R^2^ / R^2^ adjusted | 0.402 / 0.378 | | |  |  |  |

# Survey Questions and Response Scales

Table S5. Demographics and telehealth questions used in study.

| **Question** | **Response Scale** | **Waves asked?** |
| --- | --- | --- |
| Are you a U.S. military Veteran? | No (0), Yes (1) | 1 |
| What is your age? | 18 to 24, 25 to 34, 35 to 44, 45 to 54, 55 to 64, 65 to 74, 75 to 84, 85 or older | 1 |
| What is your gender? | Female (1), Male (2), Transgender woman /Transwoman (3), Transgender man /Transman (4), Non-binary/third gender (5), Prefer to self-describe (6), Prefer to not say (7) | 1 |
| What is your race? Mark all that apply. | American Indian or Alaskan Native, Asian or Asian American, Black or African American, Native Hawaiian or other Pacific Islander, White or European American, Other (please specify): text | 1 |
| How good are you at working with fractions? | Not good at all (1), 2 (2), 3 (3), 4 (4), 5 (5), Extremely Good (6) | 1 |
| How good are you at figuring out how much a shirt will cost if it is 25% off? | Not good at all (1), 2 (2), 3 (3), 4 (4), 5 (5), Extremely Good (6) | 1 |
| How often do you find numerical information to be useful? | Never (1), 2 (2), 3 (3), 4 (4), 5 (5), Very Often (6) | 1 |
| What is the highest level of schooling you have completed? (check one) | None (1), Elementary school (2), Some high school but no diploma (3), High school (Diploma or GED) (4), Some college, but no degree (5), Trade school (6), Bachelor’s degree (BS, BA, etc.) (7), Master’s degree (MA, MPH, etc.) (8), Doctoral/Professional degree (PhD, MD, etc.) (9) | 1, 3 for participants who did not see question in 1st wave |
| What is your household income? | Less than $20,000 (1), $20,000 – $29,999 (2), $30,000 - $39,999 (3), $40,000 - $49,999 (4), $50,000 - $59,999 (5), $60,000 - $74,999 (6), $75,000 - $99,999 (7), $100,000 - $149,999 (8), $150,000+ (9), Prefer to not say (10) | 1 |
| What state do you live in? | 50 States, D.C. and Puerto Rico | 1 |
| How would you best describe the place where you live? | Rural (1), Small city, e.g. less than 100,000 people (2), Suburban, near a large city (3), Mid-sized city, 100,000 to 1 million people (4), Large city, more than 1 million (5), Other, please specify (6) | 1 |
| As far as you know, do you have any of the following health conditions at the present time? - Asthma, emphysema, or chronic bronchitis, COPD (other lung disease) | Yes, I have this condition (1), No, I do not have this condition (0) | 1 |
| As far as you know, do you have any of the following health conditions at the present time? - Arthritis or rheumatism | Yes, I have this condition (1), No, I do not have this condition (0) | 1 |
| As far as you know, do you have any of the following health conditions at the present time? - Cancer, diagnosed in the past 3 years | Yes, I have this condition (1), No, I do not have this condition (0) | 1 |
| As far as you know, do you have any of the following health conditions at the present time? - Diabetes | Yes, I have this condition (1), No, I do not have this condition (0) | 1 |
| As far as you know, do you have any of the following health conditions at the present time? - Digestive problems (such as ulcer, colitis, or gallbladder disease) | Yes, I have this condition (1), No, I do not have this condition (0) | 1 |
| As far as you know, do you have any of the following health conditions at the present time? - Heart trouble (such as angina, congestive heart failure, or coronary artery disease, having a past heart attack) | Yes, I have this condition (1), No, I do not have this condition (0) | 1 |
| As far as you know, do you have any of the following health conditions at the present time? - HIV illness or AIDS | Yes, I have this condition (1), No, I do not have this condition (0) | 1 |
| As far as you know, do you have any of the following health conditions at the present time? - Kidney disease | Yes, I have this condition (1), No, I do not have this condition (0) | 1 |
| As far as you know, do you have any of the following health conditions at the present time? - Liver problems (such as cirrhosis) | Yes, I have this condition (1), No, I do not have this condition (0) | 1 |
| As far as you know, do you have any of the following health conditions at the present time? - Stroke | Yes, I have this condition (1), No, I do not have this condition (0) | 1 |
| As far as you know, do you have any of the following health conditions at the present time? - High blood pressure (hypertension ) | Yes, I have this condition (1), No, I do not have this condition (0) | 1 |
| As far as you know, do you have any of the following health conditions at the present time? - Very overweight or obese | Yes, I have this condition (1), No, I do not have this condition (0) | 1 |
| Do you have a preexisting health condition--respiratory illness, cancer, heart disease, high blood pressure, etc.--that you believe makes you more vulnerable to coronavirus? | No (0), Yes (1), Unsure (2) | 1 |
| Has your job changed as a result of COVID-19? | No (0), Yes (1) | 1 |
| Where do you get your health care? | Primarily at VA (1), Primarily at non-VA (2), About an equal mix of VA and non VA (3) | 1 |
| Which of the following is your PRIMARY source of payment for your health care (that is, the source of care that you use or rely on the MOST): | I don't have any health care insurance;  TRICARE, VA health care, or other military health care Insurance through a current or former employer or union (of yours or another family member's;  Insurance provided by an employer; Insurance purchased directly from an insurance company (by you or another family member). This would include coverage purchased through an exchange or marketplace, such as Healthcare.gov (e.g., "Obamacare");  Medicare, for people 65 and older, or people with certain disabilities, Medicaid, Medical Assistance (MA), the Children's Health Insurance Program (CHIP) or any kind of state or government-sponsored assistance plan based on income or a disability;  Indian Health Service;  Any other type of health insurance coverage or health coverage plan | 1 |
| Has it been easier or harder to get health care since the COVID-19 pandemic began? | Much harder to get health care (1), Somewhat harder to get health care (2), Same as before COVID (3), Somewhat easier to get health care (4), Much easier to get health care (5) | 1 |
| Have you received any care over the phone or through video web-based chat, instead of in-person visits, since the start of the COVID-19 pandemic? | Yes, I have received Telehealth (1), No, I have not received Telehealth (0) | 1 |
| Have you received any care over the phone or through video web-based chat, instead of in-person visits, since our last survey (which you took in the first half of December)? | Yes, I have received Telehealth since the last survey (1), No, I have not received Telehealth since the last survey (0) | 2 |
| In the last month, have you received any care over the phone or through video webbased chat, instead of in-person visits? | Yes, I have received Telehealth in the last month (1), No, I have not received Telehealth in the last month (0) | 3 |
| In the last month, how many telehealth visits have you had WITHIN VA healthcare? | 0 (0), 1 (1), 2 (2), 3 (3), 4 (4), 5 (5), 6 (6), 7 (7), 8 (8), 9 (9), 10+ (10) | 1,2,3 |
| In the last month, how many telehealth visits have you had OUTSIDE of VA healthcare? | 0 (0), 1 (1), 2 (2), 3 (3), 4 (4), 5 (5), 6 (6), 7 (7), 8 (8), 9 (9), 10+ (10) | 1,2,3 |
| Please select the type of telehealth visit(s) you had in the last month. (answers not mutually exclusive) | Mental health (depression, anxiety ); Regular health check-up; A health problem I had before COVID-19(e.g., high blood pressure, diabetes);  New health problem (something new that came up since the COVID-19 pandemic began) Concern that I had COVID-19; Attended health visit for child; Attended health visit for someone else (e.g., parent, spouse);  Other, please describe: text | 1,2,3 |
| Based on your recent experience, do you prefer in-person visits, telehealth visits, or do you have no preference? | Much prefer in person (1), Somewhat prefer in person (2), No preference (3), Somewhat prefer telehealth (4), Much prefer telehealth (5) | 1,2,3 |
| Why have you not used any telehealth care in the last month? (select all that apply to you) | I haven't needed to use telehealth;  It was hard to get an appointment for telehealth; My health care provider doesn't offer telehealth; My insurance won't cover a telehealth visit; I can't do a video call because I don't have a smart phone or a web camera; I can't do a video call because my Internet isn't good enough;  I don't feel comfortable doing a telehealth visit; I had other reasons for not doing a telehealth visit | 1,2,3 |
| Did you have family members or other loved ones take part in your recent telehealth visit(s)? | No (0), Yes (1) | 1,2,3 |
| Would the family member or other loved one have been able to go to the visit if it had been in-person? | No (0), Yes (1), Unsure (2) | 1,2,3 |
